# SLEEP AND CORTISOL IMPACT COGNITIVE OUTCOMES IN MIDLIFE HISPANIC/LATINE ADULTS AT RISK FOR ALZHEIMER’S DISEASE

**DOI:** 10.1093/geroni/igae098.3524

**Published:** 2024-12-31

**Authors:** Shanna Burke, Samantha Gonzales, Adrienne Grudzien, Celine Cambara, Alexandra Briceno, Daniel Jimenez, Stephanie Scott, Sabrina Sales Martinez

**Affiliations:** Florida International University, Miami, Florida, United States; Florida International University, Miami, Florida, United States; Florida International University, Miami, Florida, United States; Florida International University, Miami, Florida, United States; Florida International University, Miami, Florida, United States; Florida International University, Miami, Florida, United States; Florida International University, Miami, Florida, United States; Florida International University, Miami, Florida, United States

## Abstract

Sleep disturbance has been associated with an increased risk of Alzheimer’s disease (AD). Repeated stressors, including ongoing sleep disturbance, trigger persistent increases in glucocorticoid levels, which affect brain function. Various cognitive domains appear to be significantly impacted by sleep. This study examined the effect of sleep and cortisol levels on cognitive test outcomes. Hispanic/Latine adults (n=39; mean age 53 yrs (SD: 5.12)) with first-degree relatives with AD were recruited from Miami-Dade and Broward counties, FL. Cortisol was measured from saliva obtained three times in one day (waking, 30 minutes post-waking, and bedtime). Cognition was measured using the NACC Neuropsychological Battery (UDS3). Sleep was measured using wrist-worn actigraphy (ActiGraph wGT3X-BT). Multiple linear regression was performed to examine correlations between sleep, cognition, and cortisol. Average sleep duration was 6.40 (SD = 1.37) hours. Average efficiency was 92% (SD = 3%). Average sleep fragmentation was 27.79 (SD = 9.59) minutes post-sleep onset. Sleep efficiency was significantly associated with executive attention after adjusting for education and income (β = -18.73, p =.046). Sleep fragmentation was significantly associated with immediate memory (β = -.045, p =.0181). The effect of cortisol at waking on episodic memory delayed was dependent on sleep efficiency (β = 211.004, p =.031). The interaction between sleep duration and cortisol at bedtime was significantly associated with nonverbal memory (β =10.37, p =.023). The identification of modifiable risk factors for AD, including poor sleep and increased stress, will inform the development of intervention efforts to prevent or delay AD onset.

